# Identification of New Purpuroine Analogues from the Arctic Echinodermata *Pteraster militaris* That Inhibit FLT3-ITD^+^ AML Cell Lines

**DOI:** 10.3390/ijms232415852

**Published:** 2022-12-13

**Authors:** Sara Ullsten, Guillaume A. Petit, Johan Isaksson, Ida K. Ø. Hansen, Yannik K.-H. Schneider, Marte Jenssen, Chun Li, Kine Ø. Hansen

**Affiliations:** 1 Marbio, UiT—The Arctic University of Norway, N-9037 Tromsø, Norway; 2 Department of Chemistry, UiT—The Arctic University of Norway, N-9037 Tromsø, Norway; 3 Department of Pharmacy, Faculty of Health Sciences, UiT—The Arctic University of Norway, N-9037 Tromsø, Norway; 4 Norwegian College of Fishery Science, Faculty of Biosciences, Fisheries and Economics, UiT—The Arctic University of Norway, N-9037 Tromsø, Norway

**Keywords:** marine Echinodermata, *Pteraster militaris*, marine secondary metabolite, marine bioprospecting, structure elucidation, FLT3

## Abstract

Isolation of bioactive products from the marine environment is considered a very promising approach to identify new compounds that can be used for further drug development. In this work we have isolated three new compounds from the purpuroine family by mass-guided preparative HPLC; purpuroine K-M. These compounds where screened for antibacterial- and antifungal activity, antibiofilm formation and anti-cell proliferation activity. Additionally, apoptosis-, cell cycle-, kinase binding- and docking studies were performed to evaluate the mechanism-of-action. None of the compounds showed activity in antibacterial-, antibiofilm- or antifungal assays. However, one of the isolated compounds, purpuroine K, showed activity against two cell lines, MV-4-11 and MOLM-13, two AML cell lines both carrying the FTL3-ITD mutation. In MV-4-11 cells, purpuroine K was found to increase apoptosis and arrest cells cycle in G1/G0, which is a common feature of FLT3 inhibitors. Interactions between purpuroine K and the FLT3 wild type or FLT3 ITD mutant proteins could however not be elucidated in our kinase binding and docking studies. In conclusion, we have isolated three novel molecules, purpuroine K-M, one of which (purpuroine K) shows a potent activity against FLT3-ITD mutated AML cell lines, however, the molecular target(s) of purpuroine K still need to be further investigated.

## 1. Introduction

Marine invertebrates are viewed as a highly valuable source of lead compounds with potential of being developed into new medicines [1,2]. The endured interest in these compounds lies in their structural diversity and complexity and their desirable pharmacological properties [3]. As part of our ongoing effort to discover new bioactive compounds from marine invertebrates, fractions of the Echinodermata *Pteraster militaris*, collected at Kvadehuken, Svalbard, showed activity against the human melanoma cell line A2058 and the human pathogenic bacterium *Streptococcus agalactiae*. Previously, the fatty acid content of *P. militaris*, collected in Balsfjorden, Norway has been investigated [4]. This is however, to the best of our knowledge, the first investigation into the secondary metabolite content of the species. Through UHPLC-MS analysis, three compounds were identified as potential candidates to be responsible for the observed bioactivity. Following isolation and structure elucidation, the compounds were identified as members of the purpuroine family.

Compounds belonging to the purpuroine group have previously been isolated from the marine sponges *Pseudoceratina crassa* [5] and *Iotrochota purpurea* [6]. The common chemical features of the reported purpuroines are an alpha-amino acid with a trimethylated nitrogen, which is attached to a halogenated benzene ring via a C_2_H_3_ or C_3_H_5_O linker. In the herein reported purpuroines, the carboxylic terminal is methylated. Compounds similar to the purpuroines have been isolated from sponges belonging to the Verongiida order, like nakirodin A [7], aplysamine 3 and 4 [8] and others [5,9,10,11,12]. Previously, only limited bioactivity testing of the purpuroines have been conducted. However, both antibiotic activity and protein kinase inhibition have been reported [6].

Feline McDonough Sarcoma like tyrosine kinase receptor 3 (FMS-like tyrosine kinase 3 or FLT3) is a large transmembrane protein part of the receptor tyrosine kinase family. Its structure is divided in an extracellular receptor domain, a transmembrane domain followed by a juxta membrane (JM) domain and a cytoplasmic kinase domain split in two parts [13,14,15,16]. The kinase domains of FLT3 are usually kept in an inactivated state by the JM domain but upon binding of the FLT3 ligand (FL) to the receptor domain, FLT3 dimerize causing phosphorylation of tyrosine residues in the JM domain and activation loop, resulting in activation of the kinase domains [15]. Once active, FLT3 initiate multiple signal cascades including activation of the PI3K, AKT and RAS pathways and other tissues specific pathways leading to increases in cell proliferations and survival [17,18]. Mutations resulting in duplication of amino acid segments in the JM domain of FLT3, called internal tandem duplications (ITD), can lead to constitutive activation of the kinase, and of its downhill pathways [19]. FLT3 overactivation is the most common form of genetic alteration in acute myeloid leukemia (AML). While FLT3 inhibitors are clinically available, all come with serious adverse effects, including severe cardiac and hematological conditions [20]. 

**Figure 1:**
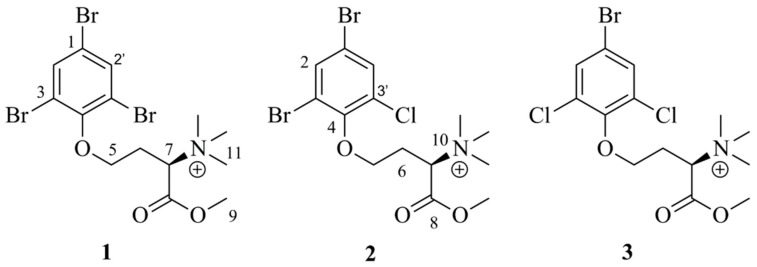
Structures of purpuroine K–M (compound **1**–**3**) isolated as part of this work.

The present study resulted in the isolation of three, previously not described, compounds belonging to the purpuroine family (Figure 1): purpuroine K (**1**), purpuroine L (**2**) and purpuroine M (**3**). Due to the previously reported bioactivity of the purpuroine family, the compounds were selected for further antibacterial and antiproliferative activity screening. Compound **1** showed selective inhibitory activity against FLT3 driven cancer cell lines. However, it did not display any direct binding to the active site of the kinase FLT3.

## 2. Results and Discussion

### 2.1. Biomass Collection, Extraction and Fractionation

*Pteraster militaris* was collected with a triangular bottom scrape off the coast of Kvadehuken, Spitzbergen (78°57′58″ N, 11°19′41″ E, wet weight = 2440.8 g). The sample was freeze-dried (dry weight = 391.3 g) and extracted with MQ-water. The resulting pellet (dry weight = 278.9 g) was re-dried and extracted twice in MeOH:CH_2_Cl_2_ (1:1). After filtration, the solvent was evaporated under reduced pressure, yielding the organic extract (43.5 g). The organic extract was fractionated into eight fractions using RP flash chromatography.

### 2.2. Viability and Antibacterial Screening of the Flash Fractions

The flash fractions of *P. militaris* were assayed for activity against the human melanoma cancer cell line A2058 at 50 µg/mL. For fraction five, this resulted in 23% cell survival. The flash fractions were also assayed against the human pathogenic bacterial strains *Staphylococcus aureus*, *Enterococcus faecalis*, *Escherichia coli*, *Pseudomonas aeruginosa* and *Streptococcus agalactiae* (Gr. B). Flash fractions three, four and five were found to be active against *Streptococcus agalactiae* at a concentration of 250 µg/mL. 

### 2.3. Dereplication of the Active Fractions

**Figure 2:**
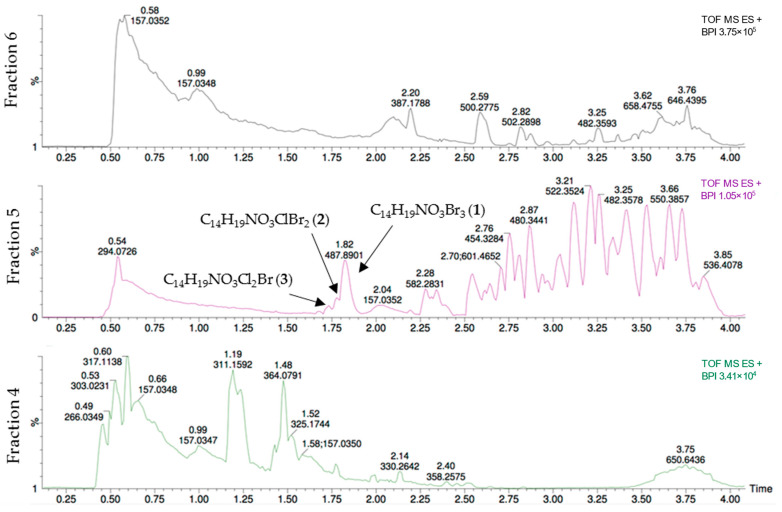
UHPLC-HR-MS chromatograms of the cytotoxic flash fraction five, as well as the non-cytotoxic fractions four and six of the organic extract of *Pteraster militaris*. Three halogenated secondary metabolites were exclusively present in flash fraction five, which suggests their involvement in the activity seen towards the human melanoma cell line A2058.

To identify the compound(s) responsible for the activity against the A2058 cell line, the active fraction five as well as the inactive fractions four and six were analyzed using UHPLC-HR-MS (Figure 2). In the chromatogram for fraction five, three halogenated secondary metabolites stood out. Their elemental compositions were calculated to be C_14_H_19_NO_3_Br_3_ (**1**), C_14_H_19_NO_3_ClBr_2_ (**2**) and C_14_H_19_NO_3_Cl_2_Br (**3**). 

The elemental compositions did not yield any hits when used as input in compound databases. As these compounds only are present in fraction five, they are likely candidates to be the compound causing the activity against the A2058 melanoma cell line and were therefore selected for isolation. In a similar manner, the chromatograms for fractions three to five were compared to fractions two and six to identify the compound(s) responsible for the antibacterial activity. In this analysis, no compound was found to be present only in the antibacterial fractions. It is possible that compounds **1**–**3** are responsible for the antibacterial activity observed for fraction five, while different compounds are responsible for the activity displayed for fractions four and three. Based on the dereplication of the fraction displaying antibacterial activity, no additional compounds were nominated for isolation.

### 2.4. Isolation of ***1**–**3***

Compound **1**–**3** were isolated from 4.06 g of the organic extract of *P. militaris* using mass guided semi-preparative HPLC. Stepwise purification using RP HPLC columns with phenyl-hexyl and C18 packing material led to the isolation of three novel purpuroine variants: purpuroine K (**1**) (4.3 mg), purpuroine L (**2**) (1.1 mg) and purpuroine M (**3**) (0.7 mg).

### 2.5. Structure Elucidation of ***1**–**3***

**Figure 3:**
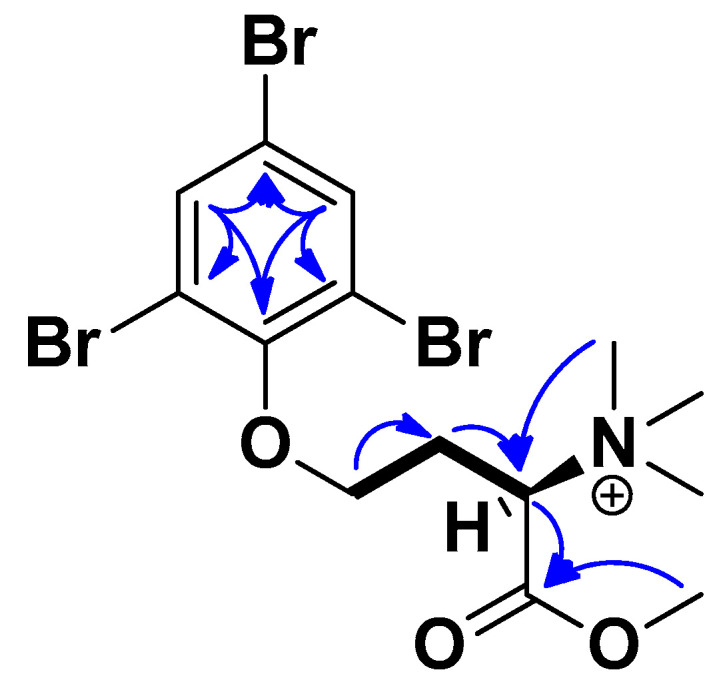
Key COSY (bold) and HMBC (blue arrows) correlations of purpuroine K (**1**).

Purpuroine K (**1**) was isolated as a light-yellow wax. HRESIMS analyses supported an elemental composition of C_14_H_19_Br_3_NO_3_ (*m*/*z* 486.8996 [M + H]^+^, calculated 486.8993), requiring five degrees of unsaturation. ^1^H and ^13^C NMR analysis (Table 1 and Table 2, Figure S1–S5) confirms the presence of 16 hydrogens and 14 carbons. 1D and 2D NMR analysis recorded for **1** revealed high structural similarity to purpuroine A (**4**) [6], with the addition of a H_3_ signal (H_3_-9) at 3.83 ppm (Figure S1) and a primary carbon (C-9) signal at 53.7 ppm (Figure S2). Through a HMBC correlation between H_3_-9 (δ_H_ = 3.83) and C-8 (δ_C_ = 167.2), the additional methyl group of **1** compared to **4** could be placed at the hydroxyl oxygen of the carboxylic acid functionality of **4**. The remaining 1D and 2D NMR data corresponded well for that recorded for **4** (Tables S1 and S2). Key COSY and HMBC correlations can be seen in Figure 3. The halogenation pattern of the benzene ring was confirmed through HSQC correlations between H-2 (δ_H_ = 7.97, 2 protons integral) and C-2 (δC = 134.0) and the lack of HSQC correlations to C-1 (δ_C_ = 117.9) and the two NMR equivalent C-3 (δ_C_ = 118.6) carbon atoms. The deshielded ppm of C-4 (δ_C_ = 151.9) confirms that the rest of the molecule is linked to this carbon via an oxygen atom. Compound **1** has one stereocenter at C-7. All the previously reported purpuroines and other N-trimethyl-D-homoserine derivates have an S-configuration in this stereocenter (initially reported as R5, but later confirmed to be S through synthesis [21]. They also all show positive signs of specific rotation. The measured [α]^20^_D_ +13.63 ± 0.02 of **1** therefore indicates the absolute configuration of C-7 to be S.

Purpuroine L (**2**) was isolated as a light brown wax. HRESIMS analyses supported an elemental composition of C_14_H_19_Br_2_ClNO_3_ (*m*/*z* 442.9414 [M + H]^+^, calculated 442.9499), requiring five degrees of unsaturation. Purpuroine M (**3**) was isolated as a light brown wax. HRESIMS analyses supported an elemental composition of C_14_H_19_BrCl_2_NO_3_ (*m*/*z* 398.9953 [M + H]^+^, calculated 399.0004), requiring five degrees of unsaturation. The ^1^H and ^13^C NMR analysis (Table 1 and Table 2, Figure S6–S15) of **2** and **3** showed close structural similarity to **1** and **4**. The core structures of **2** and **3** was solved in a similar manner as above mentioned for **1**. Compounds **2** and **3** thus have the same purpuroine backbone as **1**. When comparing the elemental compositions of **2** and **3** to the elemental composition of **1**, **2** has exchanged one bromine atom for a chlorine atom and compound **3** has exchanged two bromine atoms for chlorine atoms. For **2**, the chlorine atom was places on C-3′ (δ_C_ = 129.2) due to the downfield shift value of this carbon atom compared to the brominated carbon atoms of compound **1** (C-1: δ_C_ = 117.9, C-3/C-3′: 118.6) and the brominated carbon atoms of **2** (C-1: δ_C_ = 117.5, C-3: δ_C_ = 118.9). For **3**, the downfield shift value of C-3 and C-3′ (both δ_C_ = 129.5) compared to C-3 and C-3′ of **1** (both δ_C_ = 118.6), places the chlorine atoms in these positions. 

All the previously isolated purpuroine variants had an unmethylated carboxylic group in common. Their originating biomass, the sponge *I. purpurea*, was extracted with- and analyzed in MeOH [6]. Furthermore, sample preparation of similar compounds, like nikirodin A (with an unmethylated carboxylic acid terminal) also involved solving the biomass in methanol [7]. This indicates that the herein isolated compounds are not methylated by the extraction solvent creating artifacts of the previously reported purpuroine A, E and C, which are the unmethylated carboxylic acids of the corresponding methyl esters **1**, **2** and **3**, respectively. The sample of *P. militaris* did not contain traces of unmethylated variants of **1**–**3**.

### 2.6. Benefits to P. militaris and the Proposed Biosynthetic Origin of ***1**–**3***

The concentrations of compounds **1**–**3** were 18.88, 4.83 and 3.074 mg/kg wet weight *P. militaris*, respectively. These high concentrations indicates that they have beneficial effects for *P. militaris*. As part of this work, **1** has been shown to be cytotoxic. *P. militaris* may thus use the compounds as a defense strategy to deter predators. Some marine species have been reported to ingest organisms and sequester their secondary metabolites, using them as a chemical defense against pathogens or predators [22,23]. Species belonging to the *Pteraster* genus have been shown to primarily feed on sponges, and the herein isolated purpuroines may thus stem from a dietary source of *P. militaris* [24,25]. This is known as an acquired chemical defense strategy. As the purpuroines have been reported from sponge species that are only remotely related (*I. purpurea* and *P. crassa* belong to the same class; Demospongiae), the producer of the purpuroines may furthermore be microorganisms living in symbiosis with the sponges. The biosynthetic origin of the purpuroines remains to be determined. 

### 2.7. Antifungal, Antibacterial and Biofilm Formation Inhibiting Activity of ***1**–**3***

Due to previous documentation of antibacterial activity of compounds from the purpuroine family, compounds **1**–**3** were tested in a broad antibacterial, antifungal and biofilm formation panel. The compounds were more specifically tested for growth inhibition of the *Candida albicans* (ATCC 10231) yeast and the mould strains *Rhodotrula* sp. and the *Aurobasidium pollulans*, five pathogenic bacterial strains, *S. aureus*, *E. coli*, *E. faecalis*, *P. aeruginosa* and *S. agalactiae*, and for inhibition of biofilm formation of *S. epidermidis*. In all assays the test concentrations of the compounds were 150 µM. None of the compounds displayed activity against the microorganisms or against biofilm formation. As variants of **1** and **3** with an unmethylated carboxyl terminal previously have been reported as antifungal (IC_50_ of around 27 µM against *Aspergillus fumigatus*), this indicates that methylation of the carboxylic acid is detrimental to the antifungal activity. Furthermore, another closely related purpuroine variant (substituted at C-3 with bromine, at C-1 with hydrogen at C-5 with iodine and with a C_2_H_4_ linker and an unmethylated carboxylic acid terminal) has shown activity against *Streptococcus pneumonia*. 

While flash fractions three, four and five were found active against *S. agalactiae* at 250 µg/mL, there were no indications that **1**–**3** had growth inhibiting activities or biofilm formation inhibiting activity against any of the test strains. This indicates that a different compound present in the flash fractions was responsible for the observed antibacterial activity against *S. agalactiae*, or that a mixture of compounds produced the observed antibacterial activity. 

### 2.8. Viability Screening and IC_50_ Determination of ***1**–**3***

Anti-viability activities of **1**–**3** were evaluated in a panel of normal and cancerous cell lines at 10 µM and 50 µM (Table 3). Flash fraction five was initially active against A2058 cells. However, the isolated compounds **1**–**3** from this fraction did not show activity towards this cell line. This indicates that we did not isolate the sample component responsible for the initial observed activity, or that synergistic effects among several sample components in flash fraction five caused the cytotoxic activity. When the compounds were further studied in our full viability screening panel, **2** and **3** did not show any activity against any of the cell lines tested. However, **1** did decrease the viability of the two AML derived cell lines MV-4-11 and MOLM-13. These two cell lines are both carrying an internal tandem duplication of FLT3 (FLT3-ITD). The mutation results in autophosphorylation and constitutive activation of the receptor and multiple proliferative and survival pathways [26] leading to enhanced cell division and survival. Compound **1** seems to selectively inhibit leukocyte cell lines driven by FLT3-ITD as other leukemia derived cell lines remained unaffected suggesting inhibition of the FLT3 kinase. The active compound **1** was selected for further IC_50_ determination in the MV-4-11 cell line as well as apoptosis, cell cycle and kinase binding assays. IC_50_ of **1** against MV-4-11 was determined to be 26 ± 1.2 µM. Due to the lack of activity, **2** and **3** were not selected for further cellular or kinase binding studies.

### 2.9. Cell Cycle Analysis

**Figure 4:**
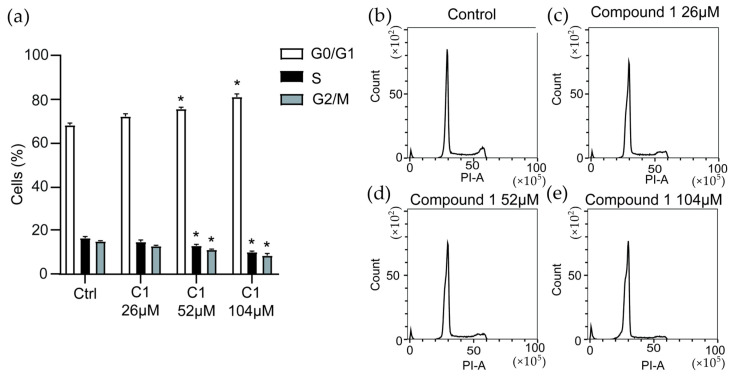
Cell cycle analysis. (**a**) MV-4-11 cells are significantly arrested in G0/G1 phase after treatment with **1** at 52 and 104 µM (2 × IC_50_ and 4 × IC_50_, respectively) with a corresponding decrease in cells in S and G2/M phase when compared to control (*n* = 3). (**b**–**e**) representative images of flow cytometry data. * denotes *p* ≤ 0.05.

To further reveal how **1** inhibits the growth of MV-4-11 cells, cell cycle analysis was performed (Figure 4). After 24 h of stimulation with **1**, significant inhibition of the cell cycle was observed at 52 µM and 104 µM compound concentration, by causing an increase in the number of cells arrested in G0/G1 phase by 11% and 19%, respectively. Parallelly a decrease of 23%, respectively, 38% of cells entering the S-phase was seen and a decrease of 26%, respectively, 55% of cells entering G2/M-phase was also observed. The progression of cells through, and exit from G0/G1, is regulated by cyclins and cyclin-dependent kinases (CDKs) [27]. Interestingly, the FLT3 kinase, which drives the proliferation of the MV-4-11 cell line, induces downstream activation of cyclin D2 and D3 which both activates CDK6 [28]. Inhibition of FLT3 has in previous studies resulted in downregulation of these cyclins which explains the common feature of G0/G1 arrest in MV-4-11 cells seen after treatment with FLT3 inhibitors [29,30]. 

### 2.10. Induction of Apoptosis

**Figure 5:**
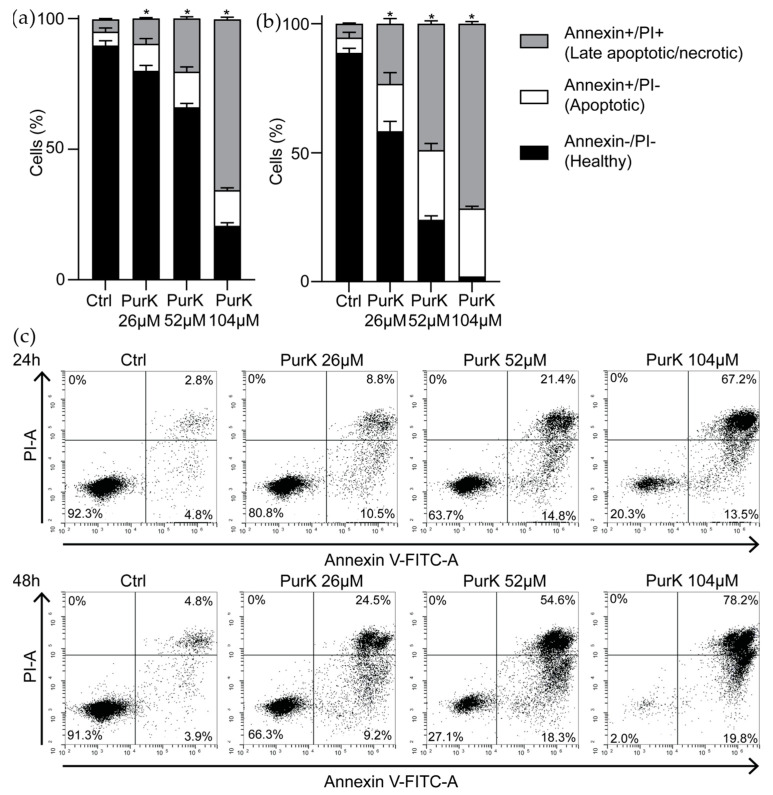
Apoptosis assay. Compound **1** (PurK) increased apoptosis in MV-4-11 cells after 24 h (**a**) and 48 h (**b**) at 26 µM, 52 µM and 104 µM compound concentration (1 × IC_50_, 2 × IC_50_ and 4 × IC_50_, respectively) when compared to control (*n* = 3). At 104 µM of compound 1, the percentages of cells in late apoptotic/necrotic phase rather than apoptotic cells increased compared to the lower concentration of 52 µM, indicating induced toxicity or that multiple targets are being affected at this concentration. Representative flow cytometry images (**c**). * denotes *p* ≤ 0.05.

To further investigate the cell death pathway activated by **1**, an apoptosis assay was performed and the ability of **1** to induce apoptosis and/or necrosis in MV-4-11 cell line was evaluated by annexin V/PI staining (Figure 5). The cells were divided into 3 different populations: annexin V−/PI− (healthy cells), annexin V+/PI− (apoptotic cells) and annexin V+/PI+ (late apoptotic/necrotic cells). After 24 h incubation, the percentage of apoptotic cells after treatment with 26 µM **1** (1 × IC_50_) were almost doubled (94% increase) when compared to control (Figure 5a,c). The induction of apoptosis was both time- and dose-dependent indicating that the target of **1** is involved in the apoptosis signaling pathway. Interestingly, FLT3 inhibitors also induce apoptosis in MV-4-11 cells which further strengthen our hypothesis that **1** targets the FLT3 kinase [31,32,33]. However, after 24 h and 48 h of incubation at the highest concentration of compound (104 µM), no increase in apoptotic cells were seen when compared to cells incubated with 52 µM. At this concentration, late apoptotic/necrotic cells instead increased significantly (Figure 5a–c). High concentrations of compound 1 does thus seem to induce necrosis over apoptosis, suggesting that multiple different targets may be affected at this concentration which is a common profile of natural products [34]. 

### 2.11. Kinase Binding and Docking

The FLT3-ITD mutant kinase is known to promote proliferative and survival pathways within the MV-4-11 and MOLM-13 cell lines. Together with selective inhibition of these two cell lines, our testing also demonstrates that MV-4-11 cells tend to arrest in the G0/G1 phase and go through apoptotic cell death upon treatment with **1**. These features are similar to FLT3 inhibitors [29,30]. In addition, some of the purpuroines reported previously displayed activity against disease relevant kinases [6]. As a results, it was decided to test the binding affinity between **1** and two FLT3 variants, namely the FLT3 wild type (WT) and FLT3-ITD mutant. 

In short, the binding between a compound of interest and the FLT3 active site can be measured with fluorescence resonance energy transfer (FRET). A FRET signal is generated between an Europium-labelled tag, on the FLT3 kinase, and a fluorescent tracer with high binding affinity for the FLT3 active site. During the assay, the compounds tested will compete against the tracer for the kinase active site, resulting in a decrease in the FRET signal based on the compound binding affinity for the kinase active site.

**Figure 6:**
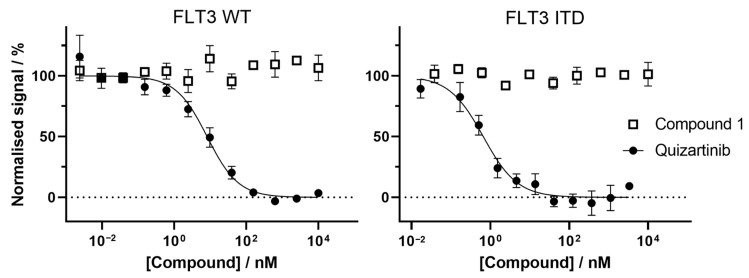
Measurement of binding between **1** and FLT3 variants. Compound **1** (squares) does not bind to either FLT3 WT or FLT-ITD mutant active site at the concentrations tested (up to 10 μM). The emission ratio (normalized signal) remains constant despite increasing compound concentration. As a comparison, quizartinib (solid circles) behave as expected, with IC_50_ of 8.2 nM against FLT3 WT and 0.7 nM against FLT3-ITD mutant, calculated from fitting the normalized signal with an inhibitory dose response curve (see methods). Error bars represent the standard deviation (*n* = 3). The *X*-axis is the Log_10_ of the compound concentrations in nanomolar (nM), and the *Y*-axis shows the normalized emission ratio signal.

Compound **1** did not cause a decrease in the FRET signal in our experimental set up, at working concentration of up to 10 μM. Since the quizartinib control worked as expected, these results suggest that **1** does not bind to the active site pocket of the FLT3 variants tested (Figure 6).

**Figure 7:**
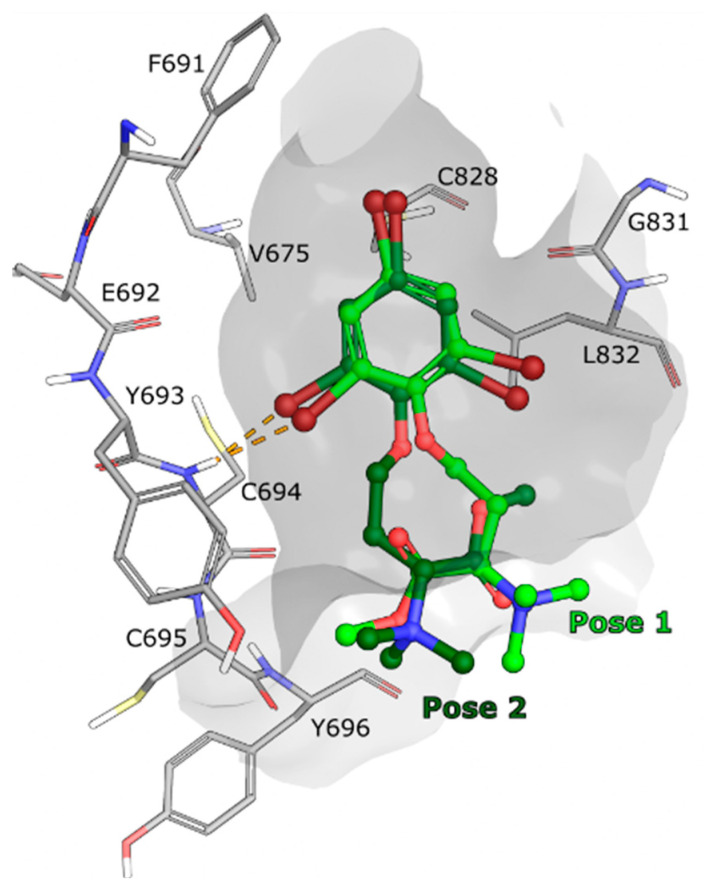
Docking of **1** in the active site of FLT3 WT model PDB ID: 6JQR [35]. Compound **1** is placed in the active site pocket (shown as a grey surface) in two different manners: pose 1 (light green carbon atoms) and pose 2 (dark green carbon atoms), with similar docking scores (both around −5.9, suggesting weak binding). Pose 2 is roughly a 180-degree rotation of pose 1 along the long axis of the molecule. However, none of the poses suggest any specific interaction with FLT3 beyond having dimensions that roughly match its active site cavity. FLT3 residues that are in proximity of **1** are displayed as grey sticks and labelled. Color code for non-carbon atoms: hydrogen atoms are shown in white, nitrogen atoms in blue, oxygen atoms in red, sulfur atoms in yellow and bromine atoms in maroon. The orange dashed lines represent too-close contacts.

In parallel to measuring the binding affinity of compound **1** for FLT3, **1**–**3** were also computationally docked in the FLT3 WT kinase domain, using the crystallographic model with Protein DataBank (PDB) ID: 6JQR [35], to evaluate their potential binding modes. Several poses were identified for compounds **1** to **3** using Glide (Schrödinger LLC, release 2022-2) [36,37,38], however none of the poses were calculated to bind strongly to the active site and no strong interaction with the ATP binding pocket of FLT3 was observed (Figure 7). As a validation of the method used, the endogenous FLT3 ligand gilteritinib, from the same crystal structure, was successfully docked back to its original position (Figure S16). 

Based on the experimental binding affinity experiment and computational docking experiments, it is unlikely that compounds **1**–**3** are binding strongly to the active site of the FLT3 variants. There is however a possibility that **1** interacts with other parts of the FLT3 kinase, that would not directly affect the active site. For example, modulating the kinase activity by interacting with the activation loop, the JM domain or even potentially with the extracellular receptor domain of FLT3, which is not present in the binding assay (only the cytoplasmic domain is used). A kinase activity assay could determine whether **1** is regulating the activity of the kinase from an allosteric site. It is also possible that compound **1** is affecting another target located downstream of the FLT3 kinase which could generate similar phenotype as a FLT3 inhibitor. While the mode of action remains to be deconvoluted, this highlights the possibility of discovering a new and selective manner to target severe and aggressive FLT3-ITD driven AML.

## 3. Materials and Methods

### 3.1. General Experimental Procedures

Optical rotations were measured on an AA-10R automatic polarimeter (Optical activity LTD, Huntingdon, UK) in MeOH. NMR spectra were acquired in DMSO-*d*_6_ on a Bruker Avance III HD (Bruker, Billerica, MA, USA) spectrometer operating at 600 MHz for protons, equipped with an inverse TCI cryo probe enhanced for ^1^H, ^13^C and ^2^H. All NMR spectra were acquired at 298 K, in 3 mm solvent matched Shigemi tubes using standard pulse programs for Proton, Carbon, HSQC, HMBC, DQCOSY and ROESY with gradient selection and adiabatic versions where applicable. ^1^H/^13^C chemical shifts were referenced to the residual solvent peak (DMSO-*d*_6_: δ_H_ = 2.50, δ_C_ = 39.51). HRESIMS were performed using a Waters LCT Premier Time-of-Flight MS with an Acquity UPLC (Waters, Milford, MA, USA), using MS grade solvents and a Waters Acquity UPLC BEH (1.7 µm, 2.1 × 100 mm) column. Compound isolation was performed using a preparative-HPLC-MS system consisting of a 600 HPLC Pump, a 2996 Photodiode Array UV detector, a 3100 Mass detector, and a 2767 sample manager (all from Waters). The following columns were used: Xselect CSH Phenyl-Hexyl Prep (5 µm, 10 × 250 mm) and SunFire. Flash chromatography was carried out using a Biotage HPFC SP4 system equipped (Biotage, Uppsala, Sweden) with a Biotage SNAP column filled with 8 g Diaion HP-20SS (Supelco Analytical, Merck, Darmstadt, Germany). All solvents used for extraction and fractionation were of HPLC grade and Milli-Q H_2_O was used. 

### 3.2. Biological Material

Individuals of the Echinodermata *Pteraster militaris* (class Asteroidea, order Velatida, family Pterasteridae) were collected with a triangular bottom scrape at Kvadehuken, Svalbard (78.9662° N, 11.3269° E) in 2011. The organism was identified by Robert A. Johansen, at the Norwegian national biobank (Marbank) and a voucher specimen (ref. M14064) was deposited in Marbank, Tromsø, Norway. The specimen was stored at −23 °C in the dark until processed.

### 3.3. Extraction and Flash Fractionation

Freeze-dried *P. militaris* (wet weight 2440.8 g) was diced and extracted twice with water at 4 °C in the dark. After supernatant removal, the remaining pellet was freeze-dried (dry weight: 391.3 g) and extracted twice in 50:50 MeOH:CH_2_Cl_2_ (vol:vol). After filtration, the solvent was pooled and evaporated under reduced pressure, and a dark red, oily residue (43.5 g) was obtained. An aliquot of the extract (1.53 g) was then fractioned using flash chromatography on a prepacked column filled with Dainon HP-20SS resin (8 g). The following step gradient was applied: MeOH/H_2_O to MeOH in five steps (5:95, 25:75, 50:50, 75:25, 100:0) followed by MeOH:acetone to acetone in two steps (50:50, 0:100) with a flow rate of 12 mL/min. A total of eight fractions were collected (72 mL each) and dried under reduced pressure.

### 3.4. Antibacterial Screening of the Flash Fractions

The flash fractions of *P. militaris* were assayed for activity against the human pathogenic bacterial strains *Staphylococcus aureus* (ATCC 25923), *Enterococcus faecalis* (ATCC 29212), *Escherichia coli* (ATCC 25922), *Pseudomonas aeruginosa* (ATCC 27853) and *Streptococcus agalactiae* (Gr. B) (ATCC 12386) at a concentration of 250 µg/mL. Briefly, suspended bacteria in log phase were added to 96-well microtiterplates at a concentration of 1.500–15.000 colony forming units/mL. The flash fractions were subsequently added and left to inoculate for 24 h before growth inhibition was observed using a 1420 Multilabel Counter VICTOR3™ (Perkin Elmer, Waltham, MA, USA) at 600 nm. Growth medium diluted with water (1:1) was used as negative control, and bacteria suspension diluted with water (1:1) as positive control. Gentamycin was used as positive assay control. 

### 3.5. Isolation of ***1**–**3***

The organic extract of *P. militaris* (4.06 g) was partitioned between 150 mL hexane and 150 mL 90% ACN three times. The pooled MeOH fractions were dried under reduced pressure and redissolved in 18 mL ACN. Aliquots of the sample were repeatedly injected onto a SUNFIRE column and eluted with a mobile phase consisting of solvents A (MQ-water and 0.1% formic acid) and B (acetonitrile and 0.1% formic acid), delivered in a gradient mode at 6 mL/min, starting from B at 10% to 58% over 16 min. The compounds were collected with retention times of **1**: 13.6 min, **2**: 13.2 min and **3**: 12.9 min. Final separation of the compounds from sample impurities and each other were achieved by an extensive series of isolation steps. Aliquots of **1**, dried and dissolved in ACN, were injected onto a phenyl-hexyl column. Compound **1** eluted after 5.4 min (gradient: 24–51.5% B over 8 min).

### 3.6. Spectroscopic Data for the Isolated Compounds

#### 3.6.1. Purpuroine K (**1**)

Light yellow wax; ${\left[\alpha \right]}_{D}^{20}$ + 13.63 (c 0.22, MeOH); ^1^H and ^13^C NMR data, see Table 1 and Table 2; HRESIMS *m*/*z* 486.8996 [M + H]^+^ (Calcd for C_14_H_20_^79^Br_3_NO_3_, 486.8993). 

#### 3.6.2. Purpuroine L (**2**)

Light brown wax; ${\left[\alpha \right]}_{D}^{20}$ 0.00 (c 0.1, MeOH); ^1^H and ^13^C NMR data, see Table 1 and Table 2; HRESIMS *m*/*z* 442.9414 [M + H]^+^ (Calcd for C_14_H_20_^79^Br_2_^35^ClNO_3_, 442.9499).

#### 3.6.3. Purpuroine M (**3**)

Light brown wax; ${\left[\alpha \right]}_{D}^{20}$ 0.00 (c 0.05, MeOH); ^1^H and ^13^C NMR data, see Table 1 and Table 2; HRESIMS *m*/*z* 398.9923 [M + H]^+^ (Calcd for C_14_H_20_^79^Br^35^Cl_2_NO_3_, 399.0004).

### 3.7. Antibacterial and Biofilm Formation Inhibiting Activity of ***1**–**3***

The three compounds (**1**–**3**) were tested for growth inhibiting abilities against five pathogenic bacterial strains. For the assay, *Staphylococcus aureus* (ATCC 25923), *Escherichia coli* (ATCC 25922) and *Pseudomonas aeruginosa* (ATCC 27853) were grown in Mueller Hinton broth (275730, Becton Dickinson and Company, Franklin Lakes, NJ, USA), and *Enterococcus faecalis* (ATCC 29212) and *Streptococcus agalactiae* (ATCC 12386) were grown in Brain Heart Infusion broth (53286, Sigma-Aldrich, St. Louis, MO, USA). The bacteria were grown on blood agar plates (University Hospital of North Norway, Tromsø, Norway) and a fresh colony was transferred to the appropriate growth media for an overnight incubation at 37 °C. The bacteria were diluted in fresh media to obtain exponential growth and reach a turbidity of 0.5 McFarland standard and added to a 96-well microtiter plate (734-2097, Nunclon™ Delta Surface, Thermo Scientific, Waltham, MA, USA). The total volume was 100 µL/well with 1500–15,000 CFU/well. The purpuroines were dissolved in Milli-Q water with 1% dimethyl sulfoxide (D4540, Sigma-Aldrich) and added to the wells in triplicates with a final concentration of 150 µM. The plate was incubated overnight at 37 °C, and the growth inhibition was measured with absorbance at 600 nm with 1420 Multilabel Counter VICTOR3™. Positive control for the assay was bacterial suspension with sterilised water (1:1) and negative control was sterilised water with growth media (1:1). A dilution series of gentamycin (A2712, Merck, Kenilworth, NJ, USA) from 32 to 0.01 µg/mL was used as a reference control for the assay. 

The biofilm formation inhibiting activity of compounds **1**–**3** against *Staphylococcus epidermidis* (ATCC 35984) was also investigated. *S. epidermidis* was grown on blood agar (University Hospital of North Norway, Tromsø, Norway) and one colony was transferred to Tryptic Soy Broth (22092, Sigma Aldrich) for overnight cultivation. The bacteria were diluted 1:100 in fresh medium with 1% dextrose (D9434, Sigma-Aldrich) to induce biofilm formation, before transferring to a 96-well Nunclon™ microtiter plate (50 µL/well). The compounds were added to the plate in triplicates, 50 µL/well giving a final volume of 100 µL/well. The purpuroines were dissolved in the same way as in the Growth Inhibition Assay and the test concentration was 150 µM for all the compounds. The plate was incubated overnight at 37 °C, and the bacteria were removed from the plate and the plate rinsed with tapped water. Before removing the bacteria, a visual examination of the plate was performed to exclude growth inhibition in any of the wells with the compounds. The biofilm formation was determined by staining with crystal violet (115940, Merck Millipore, Dramstadt, Germany). The biofilm was firstly fixated at 55 °C for one h before the biofilm was stained with 70 µL 0.1% crystal violet solution for 10 min. After incubation with crystal violet the solution was again removed, and the plate was dried for one hour at 55 °C. When the plate was dry, 70 µL of 70% ethanol (ethanol 96%, 20823.362, VWR international, Radnor, PA, USA) was added to each well and the plate was incubated with shaking for 10 min. After incubation, the presence of biofilm was measured with absorbance at 600 nm with 1420 Multilabel Counter VICTOR3™. The positive control was bacterial suspension of *S. epidermidis* with sterilised water (1:1), and the negative control was bacterial suspension of a non-biofilm forming *Staphylococcus haemolyticus* (clinical isolate 8-7A, University Hospital of North Norway, Tromsø, Norway) with sterilised water (1:1). *S. haemolyticus* was also grown in TSB with 1% dextrose and treated in the same way as *S. epidermidis*. A medium blank was also prepared with TSB with 1% dextrose and sterilised water (1:1).

### 3.8. Antifungal Activity of ***1**–**3***

The yeast strains *Candida albicans* (ATCC 10231) and *Rhodotrula* sp. and the *Aurobasidium pollulans* mold, were cultivated on potato dextrose agar with 2% glucose at room temperature. Fungal spores were dissolved in potato dextrose broth (Difco Laboratories, USA) and the cell concentration was determined and adjusted after counting in a Bürker chamber. A final fungal spore concentration of 2 × 10^5^ spores/mL were inoculated in 96-well nunc^TM^ microtiter (100 µL total well volume) plates with a final assay concentration of 150 µM of compounds **1**–**3**. The assay plates were incubated in a moist dark chamber at 37 °C. Ranging concentrations of amphotericin-B were used as positive control (MIC found to be 8.7 µM against all strains). Growth inhibition was determined microscopically after 42 h incubation.

### 3.9. Viability Screening

All cell lines were purchased from ATCC (Manassa, VA, USA) except MOLM-13 that was purchased from Leibniz Institute DSMZ (Braunschweig, Germany). Viability screening of compounds 1–3 at 10 and 50µM was performed by a colorimetric MTS assay. The cells were seeded into 96-well microtiter plates (Nunclon^TM^ Delta Surface, Thermo Scientific, Roskilde, Denmark) in RPMI-1640 supplemented with 10% FBS and 10µg/mL Gentamycin (assay media). The adherent cell lines A2058, HaCat and HT29 were seeded in a density of 2000 cells/well, MRC5 were seeded in a density of 4000 cells/well and SK-BR-3 and MDA-MB-468 were seeded in a density of 5000 cells/well. After incubation for 24 h in 37 °C and 5% CO_2_ the media was discarded and replaced by samples diluted in assay media, negative control (100% cell survival, 100% assay media) and positive control (0% cell survival, 90% assay media supplemented with 10% DMSO). The suspension cell lines MV-4-11 and MOLT-4 were directly seeded in a density of 10,000 cells/well and MOLM-13 and THP-1 were seeded in a density of 20,000 cells/well in assay media supplemented with sample. All samples were added in triplicates. Cells and samples were incubated for a total of 72 h (HepG2 cells were incubated for 24 h) at 37 °C and 5% CO_2_. Thereafter, 10% CellTiter 96^®^AQueous One Solution Reagent (G3581, Promega) containing tetrazolium compound [3-(4,5-dimethylthiazol-2-yl)-5-(3-carboxymethoxyphenyl)-2-(4-sulfophenyl)-2H-tetrazolium] and phenazine ethosulfate were added. After 1 h incubation the absorbances was measured at 485 nm with a DTX 880 and viability was calculated.

### 3.10. Cell Cycle Analysis

MV-4-11 cells were seeded into 24-well plates (Nunclon^TM^ Delta Surface, Thermo Scientific) in a density of 1 × 10^6^ cells/mL supplemented with 26 µM, 52 µM or 104 µM of **1**. The cells were incubated for 24 h and thereafter fixed in ice cold 70% EtOH. After washing the cells were stained with FxCycle™ PI/RNase Staining Solution (Invitrogen, MA, USA) according to manufacturer’s instructions. The cells were analysed by a CytoFLEX Flow cytometer (Beckman Coulter, CA, USA) and cell cycle phase determined in CytExpert software (Beckman Coulter).

### 3.11. Apoptosis Assay

MV-4-11 cells were seeded into 24-well plates (Nunclon^TM^ Delta Surface, Thermo Scientific) at a density of 1 × 10^6^ cells/mL supplemented with 26 µM, 52 µM or 104 µM of **1** and incubated for 24 or 48 h at 37 °C an 5% CO_2_. Apoptotic and necrotic cells were labelled according to manufacturer’s instructions (Alexa Fluor^TM^ 488 Annexin V/Dead Cell Apoptosis Kit, Invitrogen) and analysed by a CytoFLEX Flow cytometer and CytExpert software (Beckman Coulter).

### 3.12. Kinase Binding Assay

The FLT3 variants (FLT WT: catalogue number PV3182, lot number 2180555F; FLT ITD: catalogue number PV6191, lot number 2395171), Eu-anti-His tag antibody (catalogue number PV5596), and fluorescent tracer 236 (catalogue number PV5592) were purchased from ThermoFisher Scientific (Oslo, Norway). Anti-GST-Eu-tagged monoclonal antibodies (anti-GST AB) were purchase from Cisbio (PerkinElmer Norge AS, catalogue number 61GSTKLA) and reconstituted in water at a final concentration of 333 nM. White, non-binding, round bottom 384 well microplates (reference number 4513) were purchased from Corning (Corning Inc, Kennebunk, ME, USA). The compounds were serially diluted into DMSO to 12 different concentrations. Each of these compound-containing DMSO solutions was further diluted 33 times in assay buffer A (50 mM HEPES pH 7.5, 10 mM MgCl_2_, 1 mM ethylene glycol-bis(β-aminoethyl ether)-N,N,N′,N′-tetraacetic acid (EGTA) and 0.01% BRIJ-35) and 5 μL of these solutions were dispensed in triplicates in 384 well plate. FLT3 variants were mixed to their respective Eu tagged antibodies: FLT3 WT at 30 nM kinase concentration + 6 nM of anti- His AB and FLT3 ITD at 30 nM of kinase concentration + 12 nM of anti-GST AB. The tracer 236 was diluted to a concentration of 15 nM in buffer A. Finally, 5 μL of the kinase + antibody solution and 5 μL of the tracer solution were added to each of the wells containing 5 μL of compound in the 384 microwell plate. The plate was shaken for 1 min at 1000 rpm and incubated in the dark at room temperature for 1 h. Fluorescence measurements were made using a EnVision 2104 Multilabel reader, (PerkinElmer, US), excitation wavelength was set to 340 nm and emission was read at both 615 nm (8.5 nm bandwidth) and 665 nm (7.5 nm bandwidth) over 200 ms with a 100 ms delay between excitation and emission measurements. Emission ratio was calculated by dividing the signal at 665 nm by the signal at 615 nm for each compound and concentration. Emission ratios were normalised and plotted against the concentration and fitted with an inhibition curve (with Hill coefficient fixed to −1) of the form: (Y = 100/(1 + (X/IC_50_)) to obtain the IC_50_. Since compound **1** did not show binding to any of the FLT3 variants, the fitting of the results using this equation was poor and we decided to not display the fitting curve for compound **1** on Figure 6. 

### 3.13. Docking Studies

The compounds were imported into the Maestro Suite release 2022-2 (Maestro version 13.2.128, Schrödinger LLC, New York, NY, USA) as SMILES and prepared with LigPrep, using EPIK to simulate the protonation and tautomerisation states at pH 7.4 ± 3.0 [39,40]. After manual inspection, it was decided to use the model of the cytoplasmic kinase domain of FLT3 WT with PDB ID: 6JQR [35] for docking studies. The endogenous ligand was extracted from the protein model and prepared with LigPrep as described above. The protein model 6JQR without ligand was made ready for docking using the protein preparation wizard from Schrödinger (release 2022-2) [41]. Briefly hydrogen atoms were replaced, missing side chains and loops were repaired with PRIME [42], residues with multiple conformers were set to the conformers with the highest occupancy, all atoms were then minimised and finally water and solvent molecules were removed. A docking grid of approximate size 25 Å × 25 Å × 25 Å was generated around the active site of the prepared model, using the location of the endogenous ligand for centring. The prepared ligands were docked using GLIDE extra precision docking [36,37,38], allowing up to 3 poses per ligand and without additional constraints. Results were inspected with Maestro, figures were produced with Maestro and ChimeraX [43]. 

## 4. Conclusions

In this paper we have identified three previously undescribed purpuroines analogues, purpuroine K–M (compound **1**–**3**). The compounds did not show antifungal or antibacterial activity and did not inhibit biofilm formation. Interestingly, one of the analogues, purpuroine K (**1**), selectively inhibited the cancerous cell lines MV-4-11 and MOLM-13, two cellular models of FLT3-ITD positive human AML. Compound **1** caused cellular arrest in cell cycle phase G0/G1 and induced apoptosis in the MV-4-11 cells similar to other FLT3 inhibitors. Kinase binding and docking studies did not reveal strong binding of the compound to the FLT3 kinase and the complete mechanism behind its effects needs to be further studied. However, this indicates that it is possible to inhibit FLT3 driven AML cell lines without targeting the active site of FLT3. 

## Figures and Tables

**Table 1:** ^1^H-NMR spectroscopic data (600 MHz, DMSO-*d*_6_) for purpuroines K–M (**1**–**3**).

Position	*δ*_H_, m (*J* in Hz)		
	1	2	3
2	7.97, s	7.87, d (2.3)	7.84, s
2′		7.94, d (2.3)	
5a	4.01, m	4.08–3.95, m	4.09–4.04, m
5b			4.02–3.95, m
6a	2.74, m	2.76–2.68, m	2.71, s
6b	2.42, m	2.42, t (6.3)	2.42, d (8.0)
7	4.59, d (11.0)	4.55, dd (11.7, 2.5)	4.59–4.52, m
9	3.83, s	3.83, s	3.82, s
11	3.27, s	3.24, s	3.24, d (3.1)

**Table 2:** ^13^C-NMR spectroscopic data (150 MHz, DMSO-*d*_6_) for purpuroines K–M (**1**–**3**).

Position	*δ*_C_, Type		
	1	2	3
1	117.9, C	117.5, C	117.0, C
2	135.0, CH	132.3, CH	131.7, CH
2′		134.4, CH	
3	118.6, C	118.9, C	129.5, C
3′		129.2, C	
4	151.9, C	150.9, C	149.9, C
5	69.0, CH_2_	69.1, CH_2_	69.2, CH_2_
6	27.3, CH_2_	27.3, CH_2_	27.2, CH_2_
7	71.0, CH	71.1, CH	71.1, CH
8	167.2, C	167.2, C	167.2, C
9	53.7, CH_3_	53.6, CH_3_	53.6, CH_3_
11	51.7, CH_3_	51.7, CH_3_	51.7, CH_3_

**Table 3:** Screening of cytotoxic effect of compound **1**–**3**.

Cell Line	Cell Type	Activity at ≤50 µM ^1^		
		1	2	3
MRC5	Lung fibroblasts (non-cancerous)	I ^2^	I	I
HaCat	Keratinocytes (non-cancerous)	I	I	I
SK-BR-3	Breast cancer	I	I	I
MDA-MB-468	Breast cancer	I	I	I
MV-4-11	Acute myeloid leukemia	Active	I	I
MOLT-4	Acute lymphoblastic leukemia	I	I	I
A2058	Melanoma	I	I	I
HT29	Colon cancer	I	I	I
HepG2	Liver hepatocellular carcinoma	I	I	I
MOLM-13	Acute myeloid leukemia	Active	I	I
THP-1	Acute monocytic leukemia	I	I	I

^1^ The cytotoxic effect of compound **1**–**3** was evaluated in our in-house screening panel of cancerous and non-cancerous cell lines. Each compound was screened at 10 and 50 µM, active compound was defined as ≤50% survival at one of the concentrations. All samples were run in triplicates. ^2^ Inactive.

## Data Availability

Data available on request from the authors.

## Supplementary Materials

The supporting information can be downloaded at: https://www.mdpi.com/article/10.3390/ijms232415852/s1.
