# Supplementary material for: Identification of New Purpuroine Analogues from the Arctic Echinodermata Pteraster militaris That Inhibit FLT3-ITD+ AML Cell Lines
Source: Int J Mol Sci. 2022 Dec 13;23(24):15852. doi: 10.3390/ijms232415852 (PMC9779817; doi:10.3390/ijms232415852)
Supplement: Supplementary file 1 [file ijms-23-15852-s001.zip › ijms-2012183-supplementary.pdf]

SUPPORTING INFORMATION

Identification of new Purpuroine Analogues from the Arctic Echinodermata *Pteraster militaris* that Inhibit FLT3-ITD$^+$ AML Cell Lines

Sara Ullsten [1,*], Guillaume A. Petit [1], Johan Isaksson [2,3], Ida K. Ø. Hansen [4], Yannik K.-H. Schneider [1], Marte Jenssen [1], Chun Li [1] and Kine Ø. Hansen [1]

[1] Marbio, UiT – The Arctic University of Norway, N-9037, Tromsø, Norway
[2] Department of Chemistry, UiT – The Arctic University of Norway, N-9037 Tromsø, Norway
[3] Department of Pharmacy, Faculty of Health Sciences, UiT – The Arctic University of Norway, N-9037 Tromsø, Norway
[4] Norwegian College of Fishery Science, Faculty of Biosciences, Fisheries and Economics, UiT – The Arctic University of Norway, N-9037 Tromsø, Norway
* Correspondence: sara.m.ullsten-wahlund@uit.no Tel.: +47-77649272

Supporting Information Table of Content

NMR Spectroscopic Data for Purpuroine K (1)
Figure S1 ^{1}H NMR (600 MHz, DMSO-d_6) spectrum of purpuroine K (**1**)
Figure S2 ^{13}C (151 MHz, DMSO-d_6) spectrum of purpuroine K (**1**)
Figure S3 HSQC + HMBC (600 MHz, DMSO-d_6) spectrum of purpuroine K (**1**)
Figure S4 COSY (600 MHz, DMSO-d_6) spectrum of purpuroine K (**1**)
Figure S5 ROESY (600 MHz, DMSO-d_6) spectrum of purpuroine K (**1**)

NMR Spectroscopic Data for Purpuroine L (2)
Figure S6 ^{1}H NMR (600 MHz, DMSO-d_6) spectrum of purpuroine L (**2**)
Figure S7 ^{13}C (151 MHz, DMSO-d_6) spectrum of purpuroine L (**2**)
Figure S8 HSQC + HMBC (600 MHz, DMSO-d_6) spectrum of purpuroine L (**2**)
Figure S9 COSY (600 MHz, DMSO-d_6) spectrum of purpuroine L (**2**)
Figure S10 ROESY (600 MHz, DMSO-d_6) spectrum of purpuroine L (**2**)

NMR Spectroscopic Data for Purpuroine M (3)
Figure S11 ^{1}H NMR (600 MHz, DMSO-d_6) spectrum of purpuroine M (**3**)
Figure S12 ^{13}C (151 MHz, DMSO-d_6) spectrum of purpuroine M (**3**)

Figure S13 HSQC + HMBC (600 MHz, DMSO-d_6) spectrum of purpuroine M (**3**)
Figure S14 COSY (600 MHz, DMSO-d_6) spectrum of purpuroine M (**3**)
Figure S15 ROESY (600 MHz, DMSO-d_6) spectrum of purpuroine M (**3**)

Table S1 Experimental ^{1}H-NMR shift values of purpuroine K (**1**) compared to shift values for the previously reported purpuroine A (**4**)
Table S2 Experimental ^{13}C-NMR shift values of purpuroine K (**1**) compared to shift values for the previously reported purpuroine A (**4**)
Figure S16 Validation of the docking parameters using the endogenous ligand gilteritinib from crystal structure of the kinase domain of FLT3 with PDB ID: 6JQR

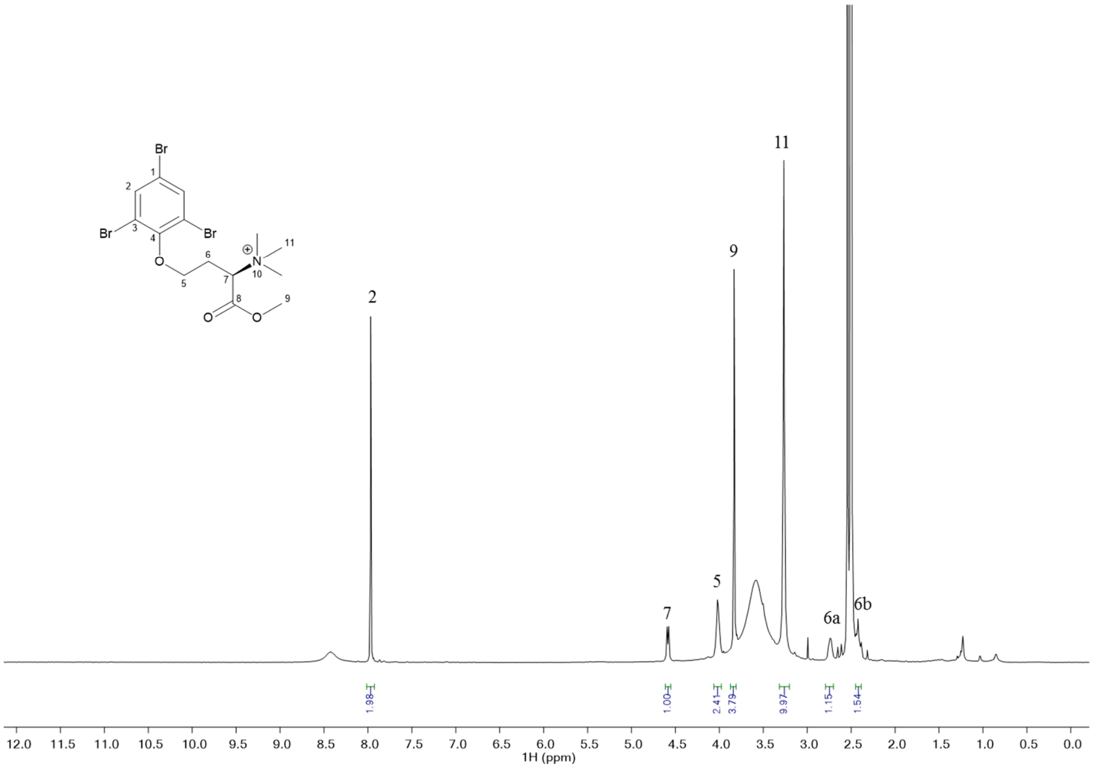

Figure S1. ^{1}H NMR (600 MHz, DMSO-d_6) spectrum of purpuroine K (**1**).

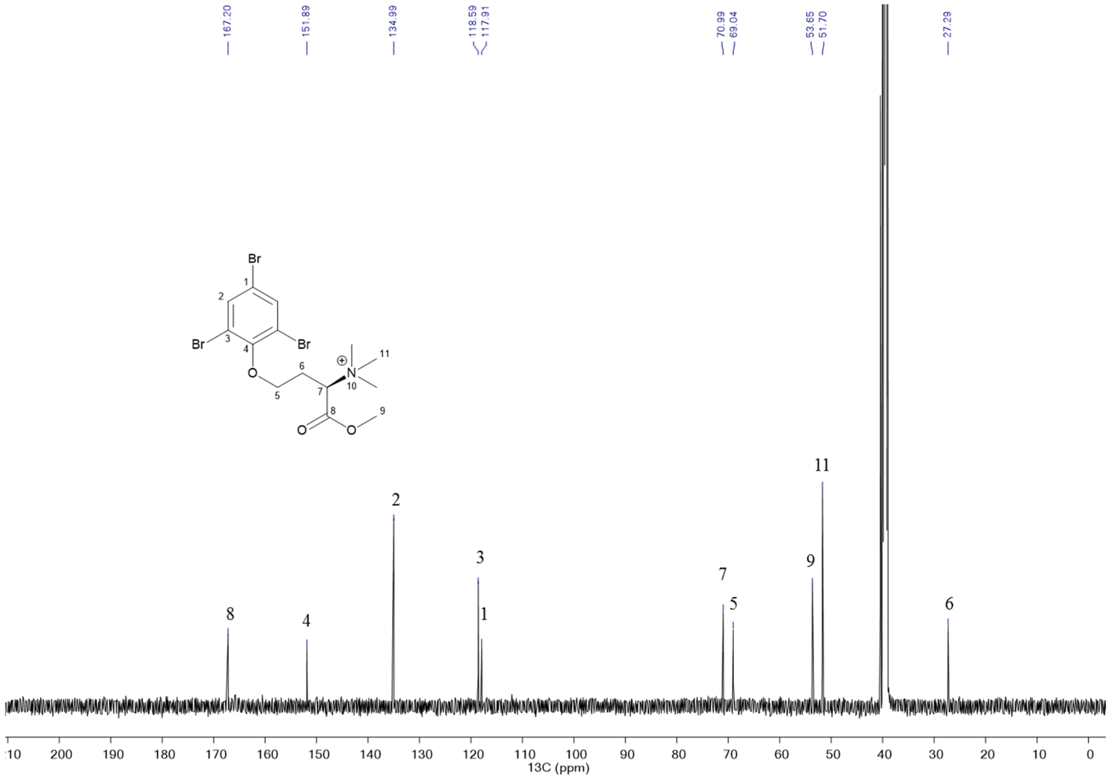

Figure S2. ^{13}C (151 MHz, DMSO-d_6) spectrum of purpuroine K (**1**).

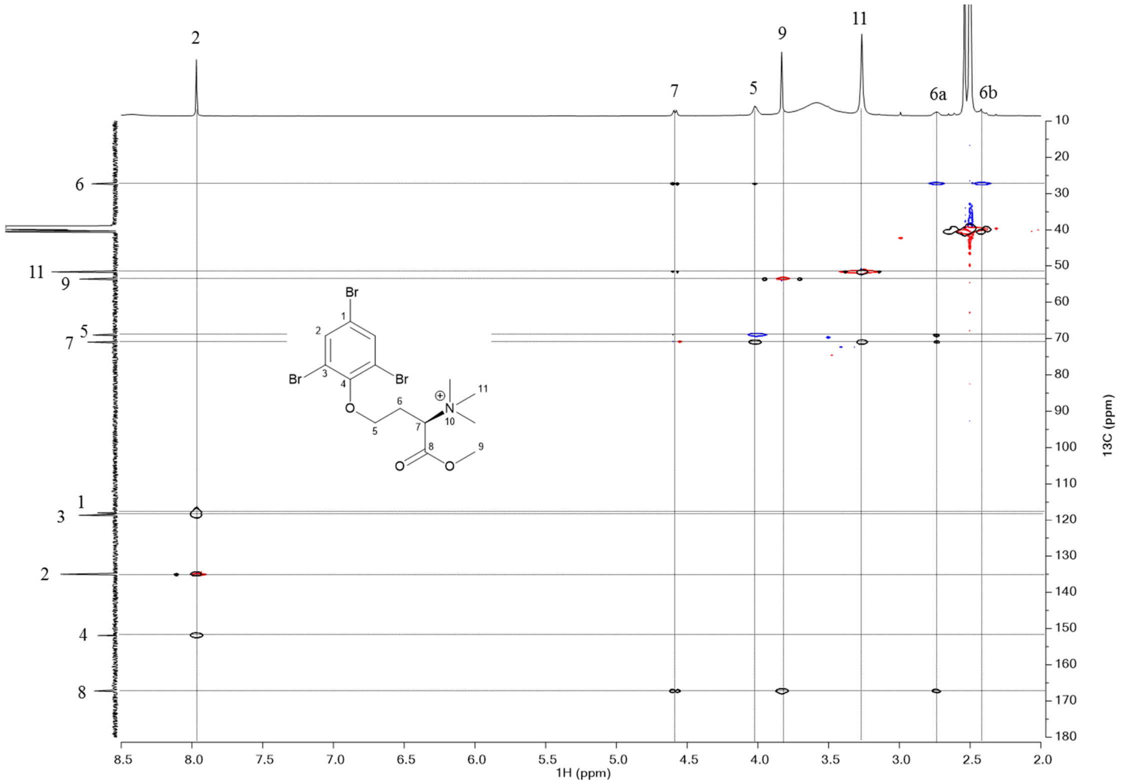

Figure S3. HSQC + HMBC (600 MHz, DMSO-d_6) spectrum of purpuroine K (**1**).

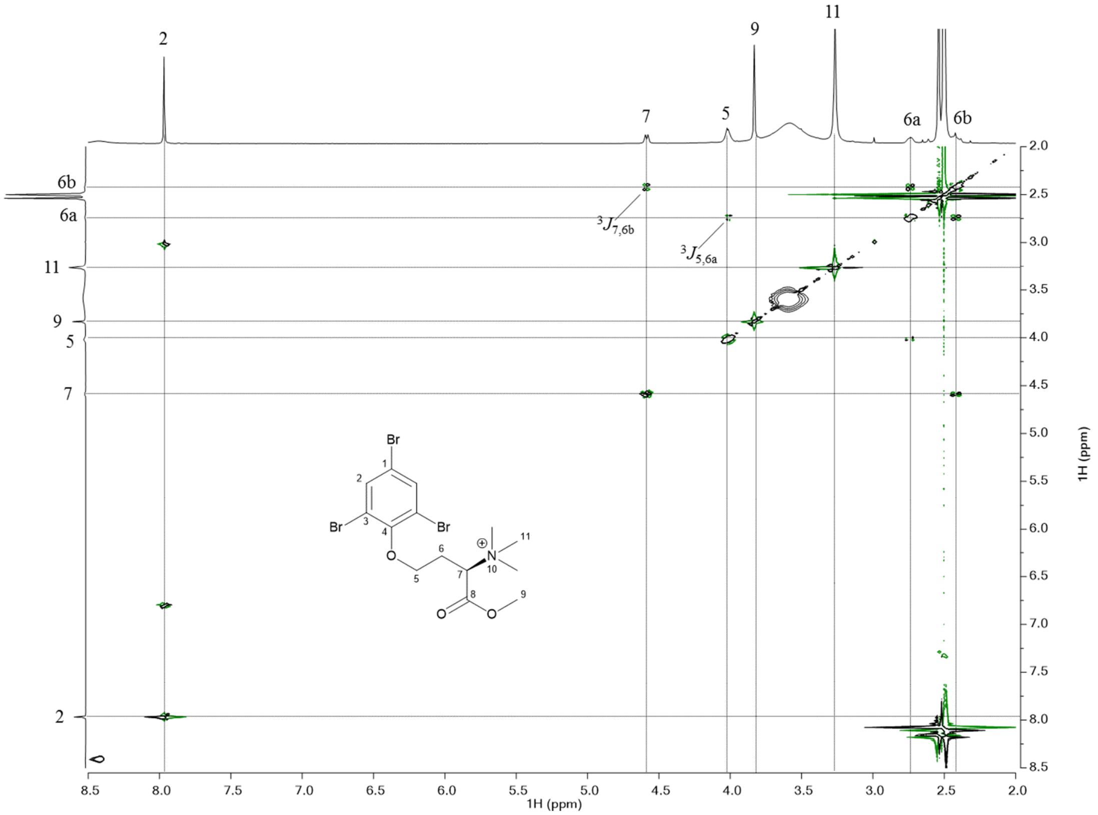

Figure S4. COSY (600 MHz, DMSO-d_6) spectrum of purpuroine K (**1**).

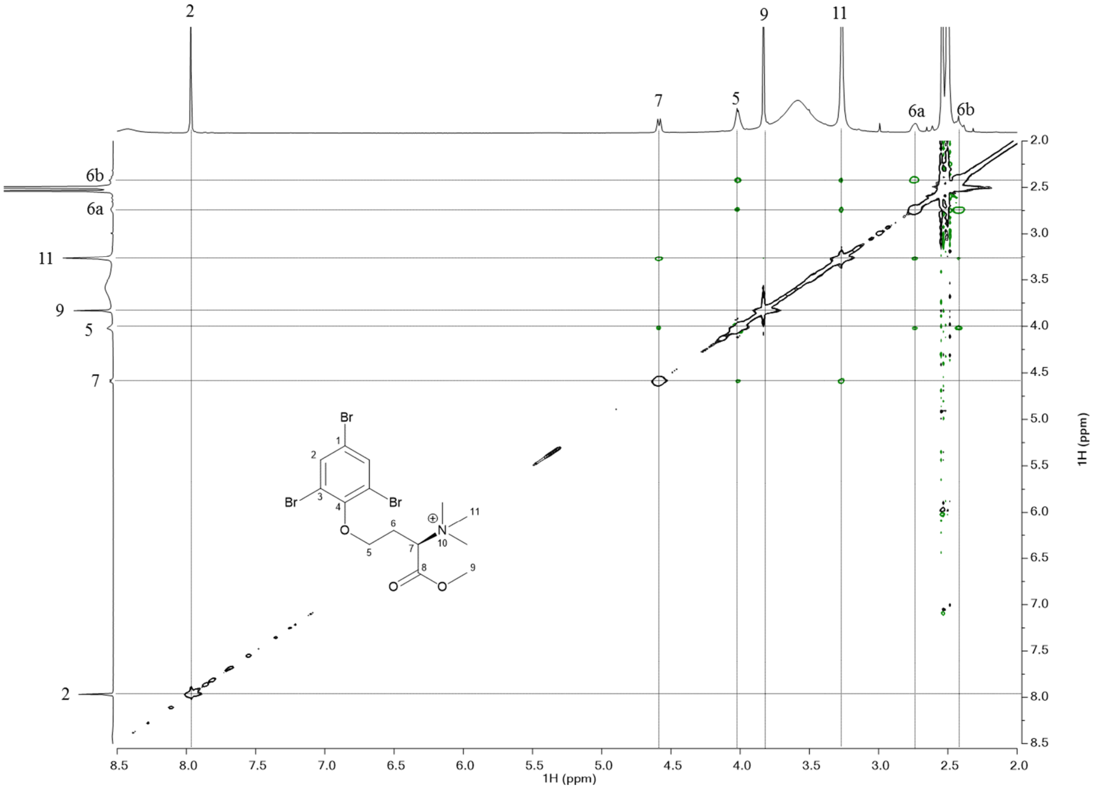

Figure S5. ROESY (600 MHz, DMSO-d_6) spectrum of purpuroine K (**1**).

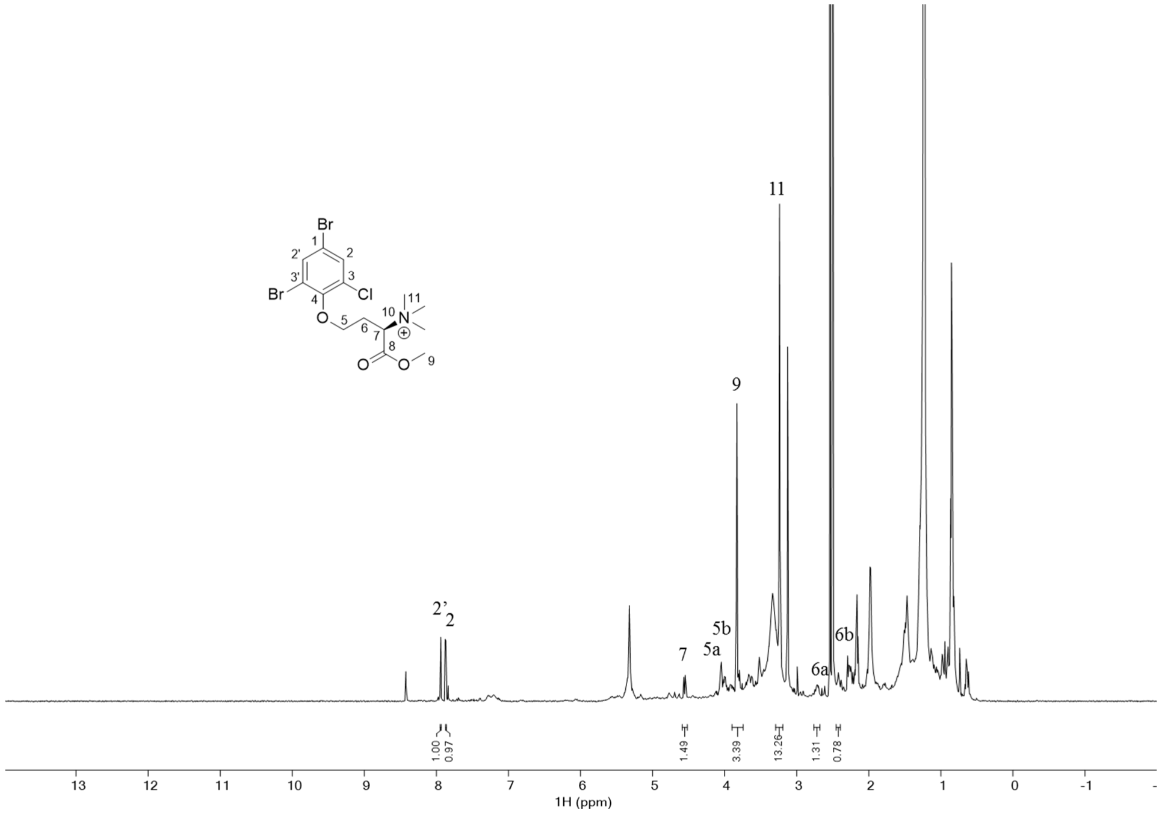

Figure S6. ^{1}H NMR (600 MHz, DMSO-d_6) spectrum of purpuroine L (**2**).

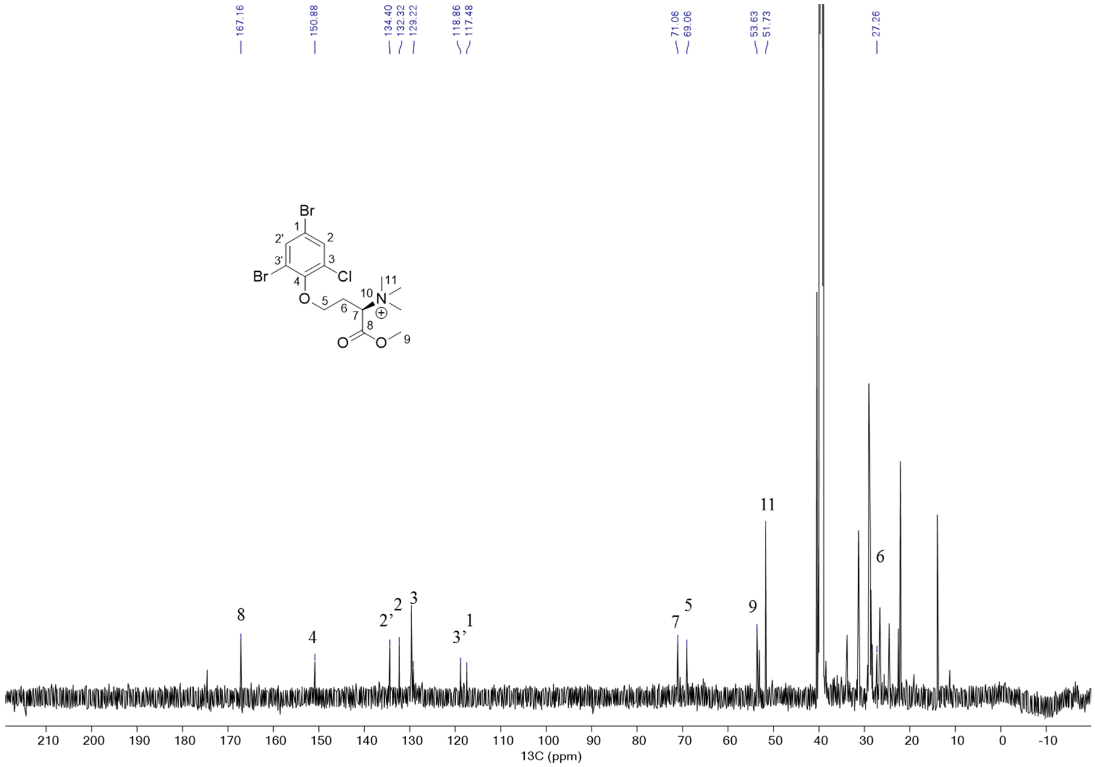

Figure S7. ^{13}C (151 MHz, DMSO-d_6) spectrum of purpuroine L (**2**).

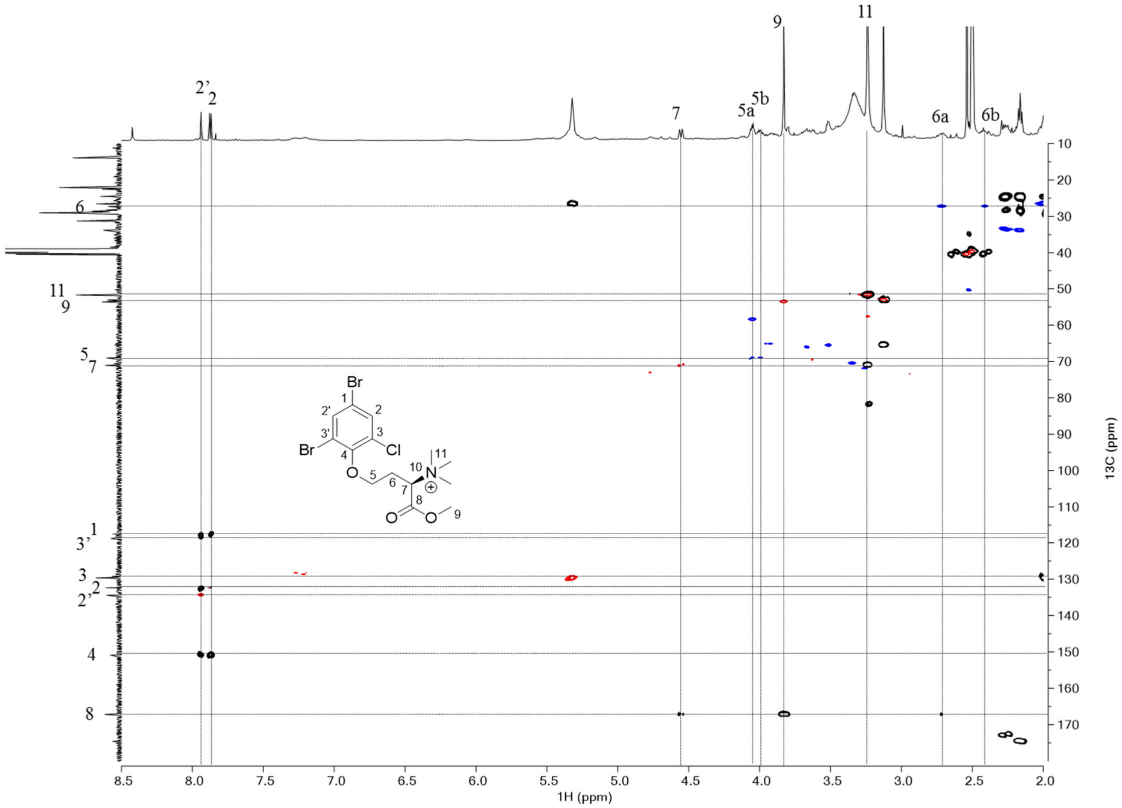

Figure S8. HSQC + HMBC (600 MHz, DMSO-d_6) spectrum of purpuroine L (**2**).

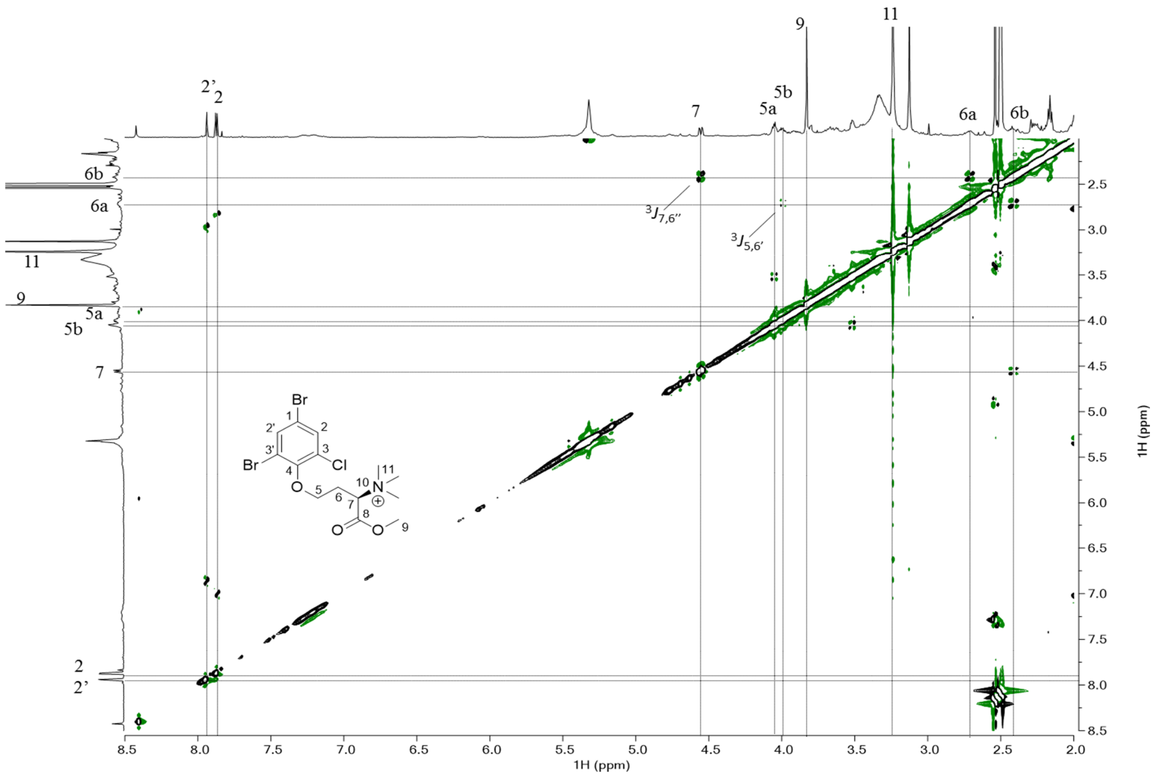

Figure S9. COSY (600 MHz, DMSO-d6) spectrum of purpuroine L (**2**).

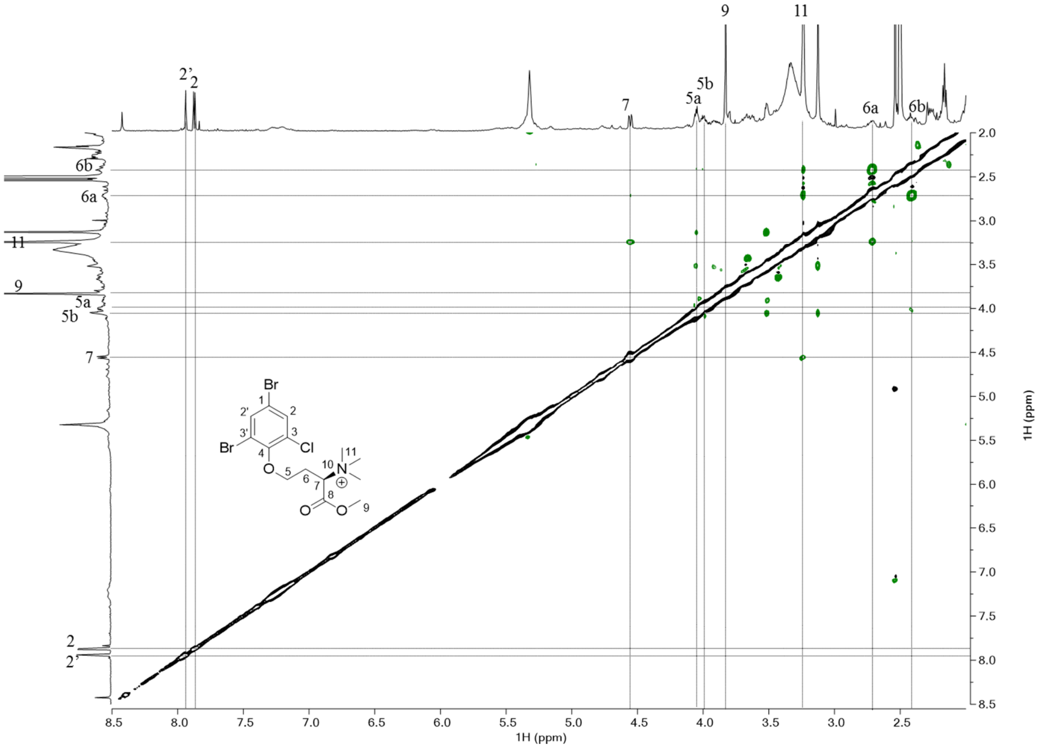

Figure S10. ROESY (600 MHz, DMSO-d_6) spectrum of purpuroine L (**2**).

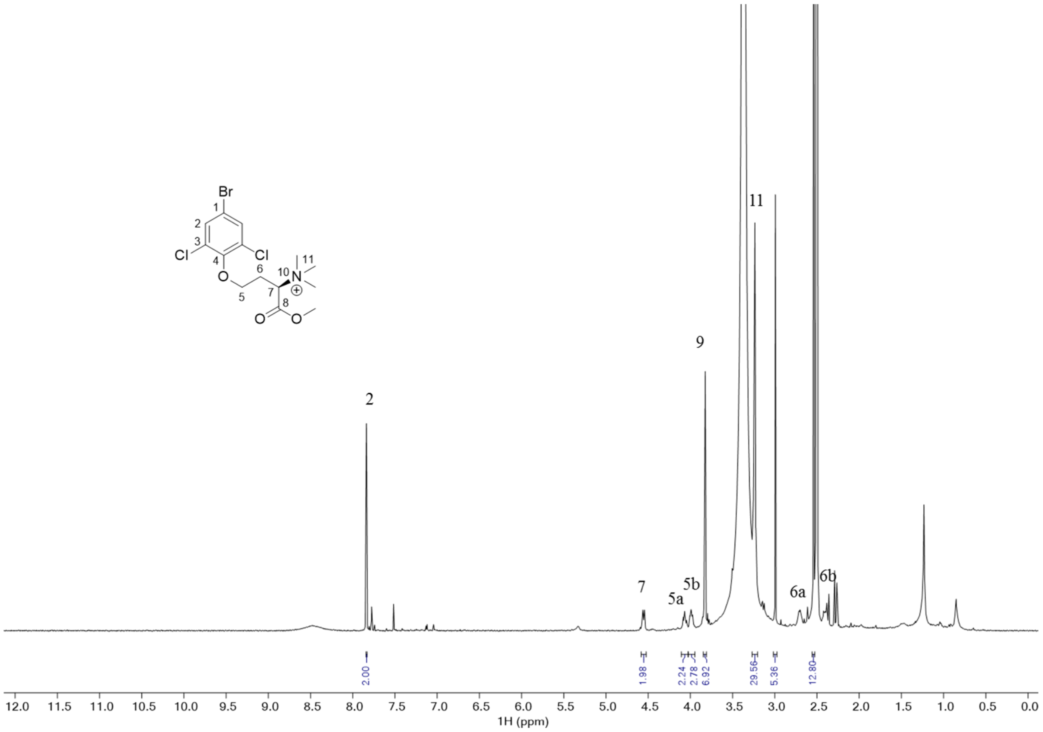

Figure S11. ^{1}H NMR (600 MHz, DMSO-d_6) spectrum of purpuroine M (**3**).

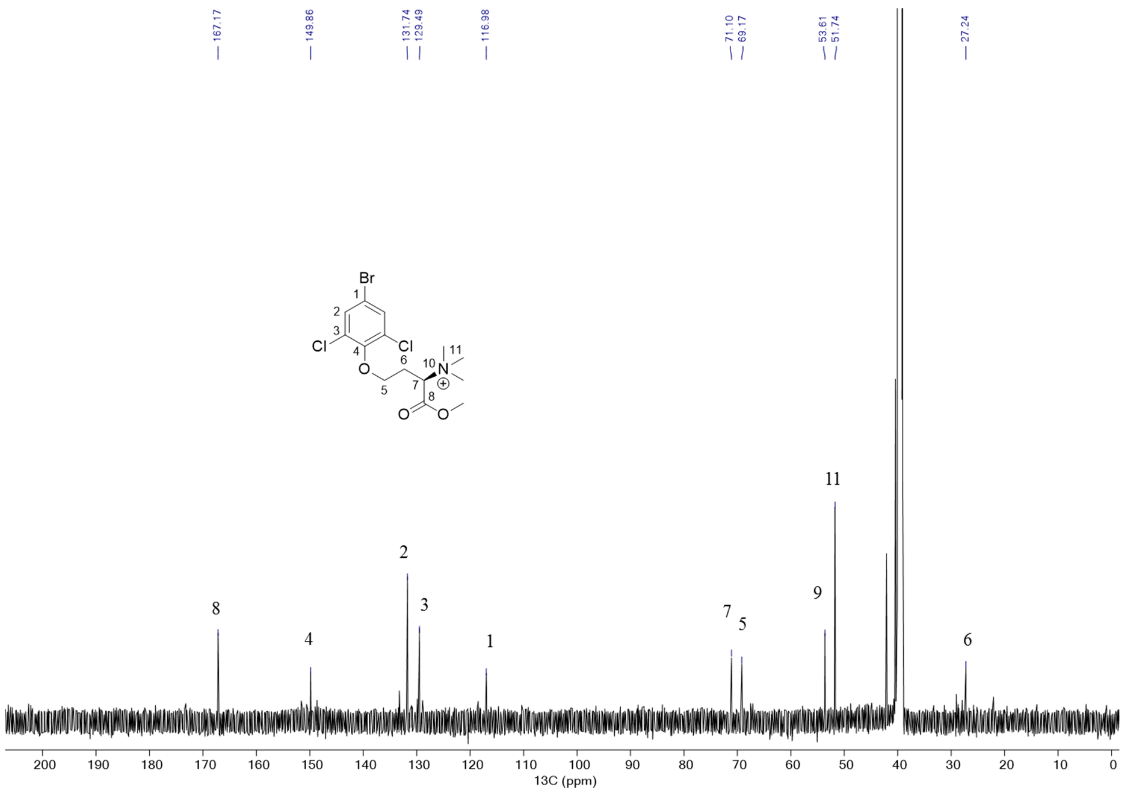

Figure S12. ^{13}C (151 MHz, DMSO-d_6) spectrum of purpuroine M (**3**).

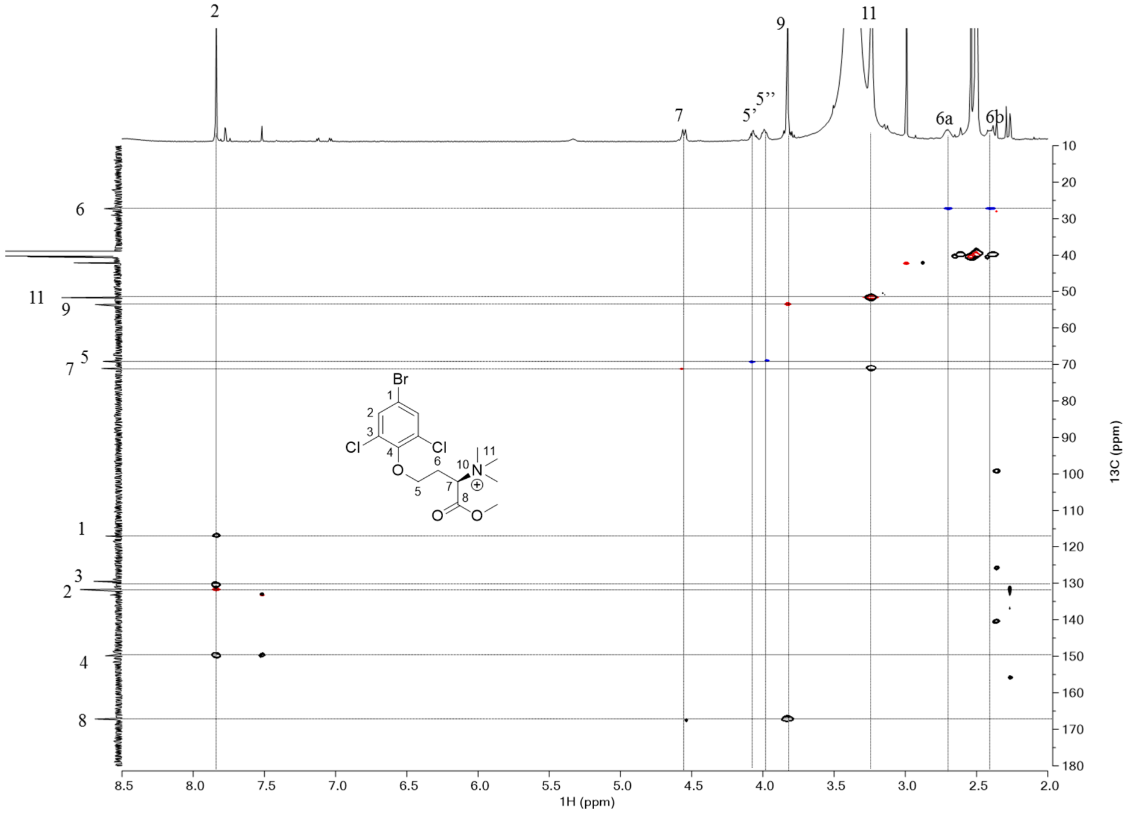

Figure S13. HSQC + HMBC (600 MHz, DMSO-d_6) spectrum of purpuroine M (**3**).

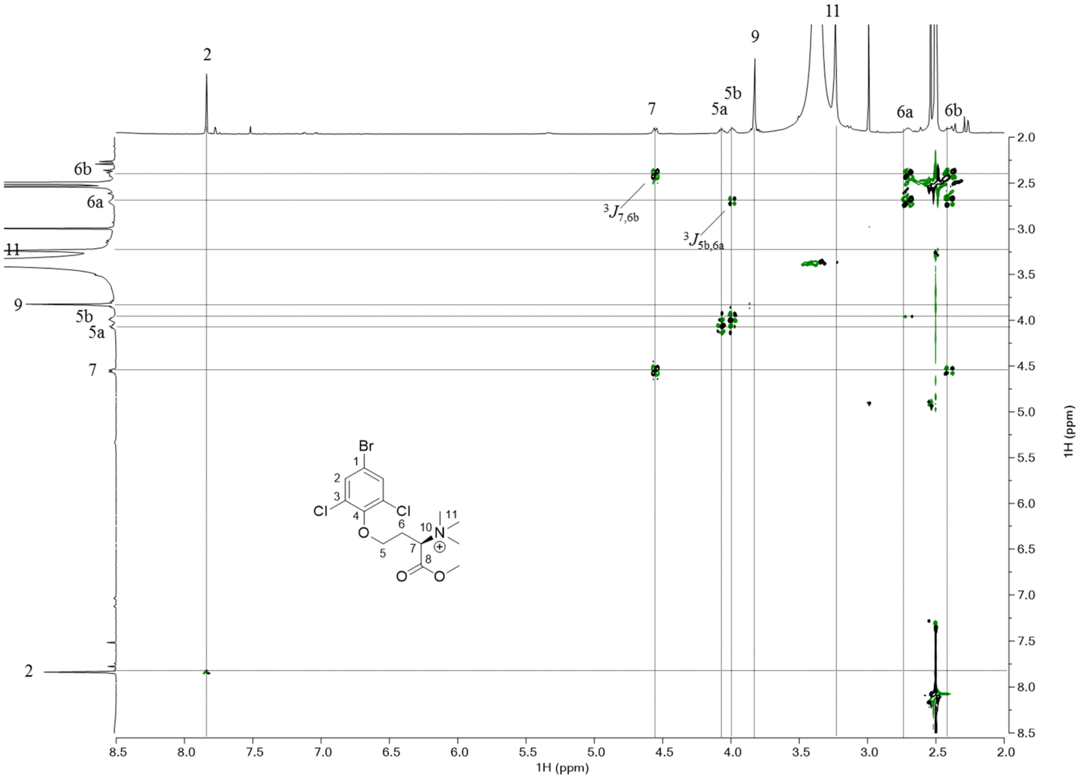

Figure S14. COSY (600 MHz, DMSO-d_6) spectrum of purpuroine M (**3**).

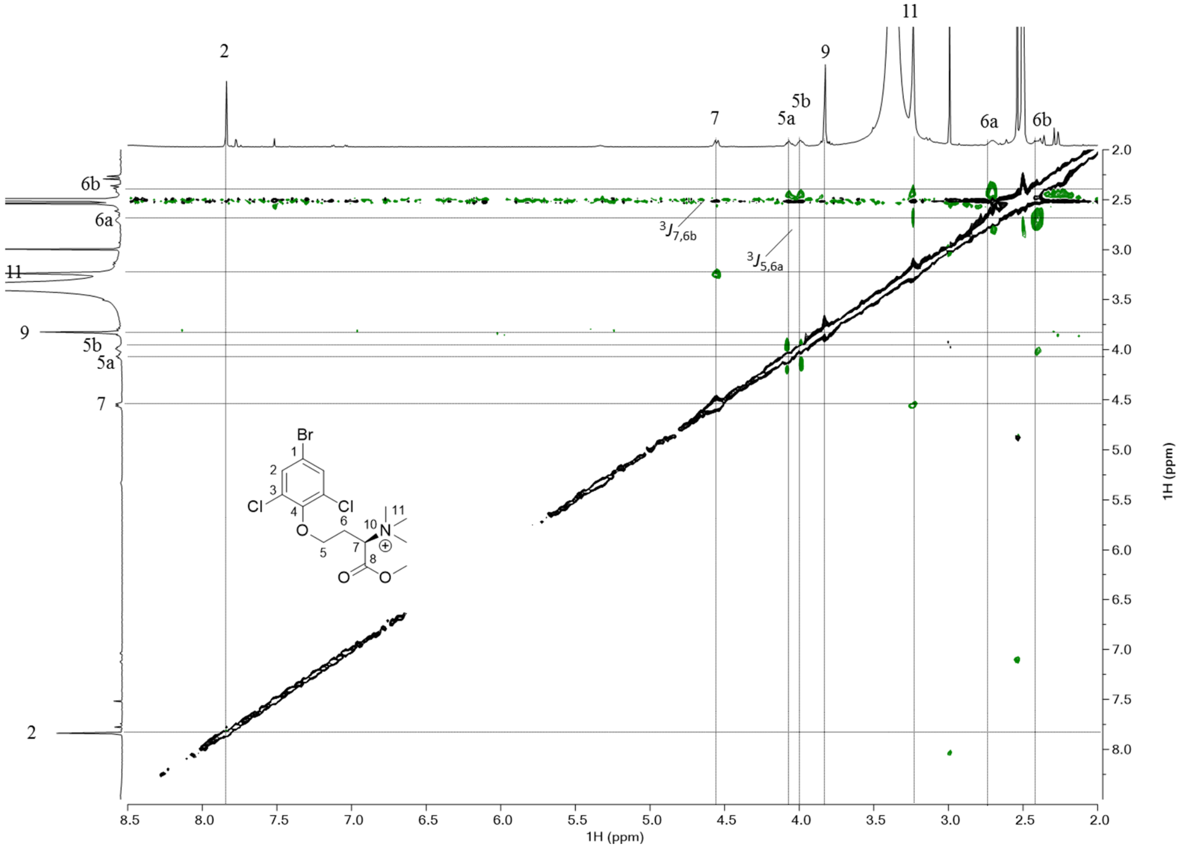

Figure S15. ROESY (600 MHz, DMSO-d_6) spectrum of purpuroine M (**3**).

Table S1. Experimental ^{1}H-NMR shift values of purpuroine K (**1**) compared to shift values for the previously reported purpuroine A (**4**).

	**δ_H, m (*J* in Hz)**	
Position	Purpuroine K (**1**)	Purpuroine A (**4**)
2<br>2'	7.97, s	7.95, s
5a	4.01, m	4.12, dd (15.8, 8.4)
5b		4.00, ddd (13.8, 8,7, 5.0)
6a	2.74, m	2.41, m
6b	2.42, m	2.20, m
7	4.59, d (11.0)	3.58, dd (11.3, 2.3)
9	3.83, s	-
11	3.27, s	3.16, s

Table S2. Experimental ^{13}C-NMR shift values of purpuroine K (**1**) compared to shift values for the previously reported purpuroine A (**4**).

	**δ_C, type**	
Position	Purpuroine K (**1**)	Purpuroine A (**4**)
1	117.9, C	117.8, C
2	135.0, CH	135.3, CH
2'		135.1, CH
3	118.6, C	119.1, C
3'		
4	151.9, C	152.9, C
5	69.0, CH_2	72.0, CH_2
6	27.3, CH_2	28.2, CH_2
7	71.0, CH	74.8, CH
8	167.2, C	166.9, C
9	53.7, CH_3	-
11	51.7, $(CH_3)_3$	51.1, $(CH_3)_3$

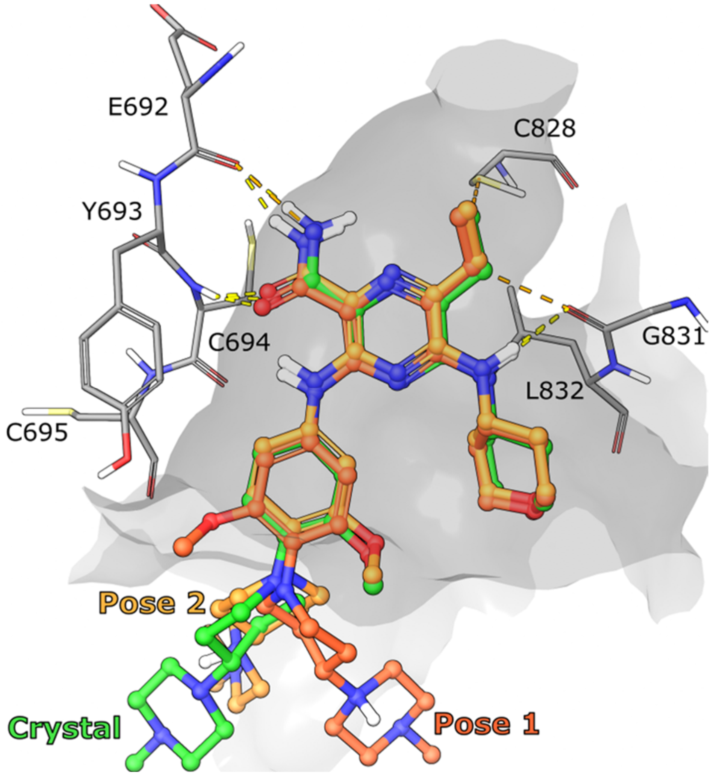

Figure S16. Validation of the docking parameters using the endogenous ligand gilteritinib from crystal structure of the kinase domain of FLT3 with PDB ID: 6JQR [1]. The ligand gilteritinib was extracted from its crystallographic model, prepared with LigPrep with the other compounds, as described in the method section, and docked back into the model as described for the other compounds. Pose 1 and 2 (in shades of orange) show the docking results whereas the original position of gilteritinib from crystallisation experiment is shown in green and labelled crystal. The docking poses for the part of the ligand found inside the active site pocket (shown as grey surface) are almost identical to the crystallographic results, in particular hydrogen bonds to the residues Glu692, Cys694 and Gly831 are conserved (shown as yellow dashed lines). The position of the ligand outside of the active site pocket varies more as there are less options for specific interactions. The docking scores for the two poses presented are - 10.8 respectively - 10.7. Relevant residues in the kinase active site are shown as grey sticks and labelled.

1. Kawase, T.; Nakazawa, T.; Eguchi, T.; Tsuzuki, H.; Ueno, Y.; Amano, Y.; Suzuki, T.; Mori, M.; Yoshida, T. Effect of Fms-like tyrosine kinase 3 (FLT3) ligand (FL) on antitumor activity of gilteritinib, a FLT3 inhibitor, in mice xenografted with FL-overexpressing cells. *Oncotarget* **2019**, *10*, 6111-6123, doi:10.18632/oncotarget.27222.
